# Trigeminal nerve electrophysiological findings in hemifacial atrophy: A systematic literature review and retrospective chart review

**DOI:** 10.1016/j.cnp.2020.12.003

**Published:** 2021-01-23

**Authors:** Michael P. Skolka, Lisa A. Marks, Lyell K. Jones, Megha M. Tollefson, Jonathan H. Smith

**Affiliations:** a Department of Neurology, Mayo Clinic, Rochester, MN, USA; b Department of Library Services, Mayo Clinic, Scottsdale, AZ, USA; c Department of Dermatology, Mayo Clinic, Phoenix, AZ, USA; d Department of Neurology, Mayo Clinic, Phoenix, AZ, USA

**Keywords:** Hemifacial atrophy, Parry-Romberg, Trigeminal nerve, Literature review, Evoked potential, And electromyography

## Abstract

**Objective:**

Hemifacial atrophy (HFA) is a rare disorder characterized by progressive unilateral wasting facial soft tissue, muscle, and/or bone. Trigeminal nerve abnormalities may contribute to or result from disease pathophysiology. We aimed to gain further insights into the role of trigeminal pathophysiology along the HFA severity spectrum.

**Methods:**

A systematic literature review was performed according to PRISMA standards. Retrospective cases of HFA from the literature and Mayo Clinic EMG database were pooled for descriptive and semi-quantitative analysis.

**Results:**

Overall, 13 total HFA patients were identified through literature and database reviews. Trigeminal nerve testing was abnormal in 9/13 (69%), exclusively in moderate-severe cases. Abnormalities suggested a peripheral (7/9, 78%) or mixed central/peripheral (2/9, 22%) localization. Trigeminal nerve abnormalities were not identified in any of the 4 cases with mild disease severity.

**Conclusion:**

Moderate to severe cases of HFA were associated with electrophysiological trigeminal abnormalities. No abnormalities were seen in mild cases of HFA.

**Significance:**

Trigeminal nerve electrophysiology may serve as a biomarker of moderate-severe disease progression, likely reflecting the consequences of progressive soft tissue atrophy.

## Highlights

• Trigeminal nerve electrophysiology is commonly abnormal in cases of HFA.
• Trigeminal abnormalities are seen in cases with moderate-severe disease.
• Both central and peripheral nervous system abnormalities may result in HFA.

## 1 Introduction

Hemifacial atrophy (HFA), otherwise known as Parry-Romberg Syndrome (PRS), is a rare disorder that involves chronic progressive wasting predominantly of hemifacial soft tissue, muscle, and somtimes bone (Falla et al., 2012). Neurological comorbidities associated with HFA include facial neuropathic pain (Viana et al., 2011, Kumar et al., 2009), headache (Amaral et al., 2013), and epilepsy (English et al., 2018). The syndrome is frequently associated with linear morphea/en coup de sabre and is highly recognizable despite lacking consensus diagnostic criteria. The pathophysiology remains elusive, although trauma, autoimmune disease, and infection have all been theorized as potential causes (Tolkachjov et al., 2015).

The chronic progressive course typically beginning at a young age and could suggest a degenerative mechanism. Previously, sympathetic dysfunction has been implicated based on case reports (Lonchampt et al., 1995), as well as experimental observations of superior cervical ganglion ablation in animals recapitulating the features of HFA (Resende et al., 1991). Upregulation of pro-inflammatory and pro-apoptotic gene pathways have been found in biopsied skin samples, as well as downregulation of genes important for the integrity and maintenance of the extracellular matrix (Chen et al., 2018). In patients presenting with early HFA, the differential diagnosis of trigeminal motor neuropathies (Condie et al., 2018), such as facial-onset sensory and motor neuronopathy (FOSMN) should be considered (Zhang et al., 2019), although HFA is unique in progression to involve soft tissue atrophy and absence of progression to a more diffuse neurological disorder. However, it should be noted that facial and limb myopathic changes may be under-recognized, as highlighted by one small case series of individuals at least with isolated linear scleroderma (Saad Magalhães et al., 2014).

Given the restricted hemifacial topography of the disorder and frequent cutaneous manifestations, we hypothesized that characterization of trigeminal nerve electrophysiology in HFA might provide additional pathophysiological insight. Evaluation of the trigeminal pathways in HFA appear to be limited to individual case reports, describing both peripheral and central abnormalities (Lonchampt et al., 1995, Budrewicz et al., 2012). However, abnormalities in trigeminal neurophysiology have not been universally reported (Falla et al., 2012).

To elucidate the potential role of trigeminal neurophysiology in patients with HFA, we performed a systematic literature review to identify studies reporting the results of trigeminal nerve testing in HFA patients. In addition, we aimed to further contribute to the literature by reporting the retrospective Mayo Clinic experience with trigeminal electrophysiological studies in HFA patients. *A priori,* our hypothesis was that trigeminal nerve abnormalities would be common in patients with HFA, and that early trigeminal nerve involvement would suggest a role in HFA pathophysiology. Further, we hypothesized that early trigeminal nerve involvement might serve as a sensitive diagnostic biomarker to help facilitate early HFA recognition.

## 2 Methods

The study was deemed exempt by Mayo Clinic’s Institutional Review Board.

For the literature review, the following databases were searched: Ovid EMBASE (1988 to 2020 March 05), Ovid MEDLINE 1946 to present), Scopus (1960 to present) and Web of Science (1975 to 2020). In all databases, the following MeSH terms were searched: “Trigeminal Nerve Diseases”, “Trigeminal Nerve”, and “Facial Hemiatrophy”. The following key/text words were also searched in each database: “jaw jerk”, “blink reflex”, “hemifacial atrophy”, “hemi-facial atrophy”, “parry romberg syndrome”, “parry-romberg syndrome”, “neuropath*”, and “trauma”. Terms were combined using the Boolean operators of “AND” and “OR” to create the search strategy which generated 53 references, limited to English language. Of the total citations, 14 duplicates were removed leaving 39 references for inclusion or exclusion review.

In accordance with the standards of PRISMA (Moher et al., 2009), two independent reviewers screened the 39 articles and removed articles that were not pertaining to HFA leaving 30 articles for further analysis. The reviewers then assessed each of the remaining 30 articles for content and excluded articles if they did not include patients who were diagnosed with HFA, if the included patient’s symptoms were from a secondary cause (e.g. tumor), or if the article did not contain trigeminal electrophysiological data.

For the retrospective chart review, patients diagnosed with HFA at Mayo Clinic Rochester and Arizona were identified January 1, 2000 to May 28, 2020 via searching the Mayo Clinic EMG database as well as from a previously identified case list from one of the investigators (MT). A total of 82 patients diagnosed with HFA were identified, and their charts were manually reviewed for study inclusion. Case inclusion required diagnosis of HFA at any age and report of trigeminal nerve electrophysiological data. Cases were excluded with alternative diagnoses or confounding comorbidities, such as facial trauma or deformity, or histories of trigeminal and/or facial neuropathies, including Bell’s Palsy. The following information was abstracted: sex, age of onset, side of face affected, symptom severity graded on the surgical scale (Raposo-do-amaral et al., 2014), associated neurological and non-neurological symptoms, time of symptom duration to electrophysiological testing, associated images including CT head and MRI brain scans, blink reflex testing, nerve conduction studies, and EMG needle studies. The information was detailed and analyzed with descriptive statistics for further review.

For grading severity of patient symptoms, a HFA severity grading scale was used which was originally designed as a scale to determine optimal surgical approach to hemifacial reconstruction in HFA (Raposo-do amaral et al., 2014). Patients in this report are described as having either mild, moderate, or severe symptoms. Mild symptoms indicate the patient’s atrophy involved only the skin, i.e. epidermis, dermis, and/or subcutaneous tissues. Moderate grading of symptoms includes atrophy of skin plus facial muscles. Severe symptoms include atrophy of the skin plus muscle plus underlying bone.

## 3 Results

**Fig. 1:**
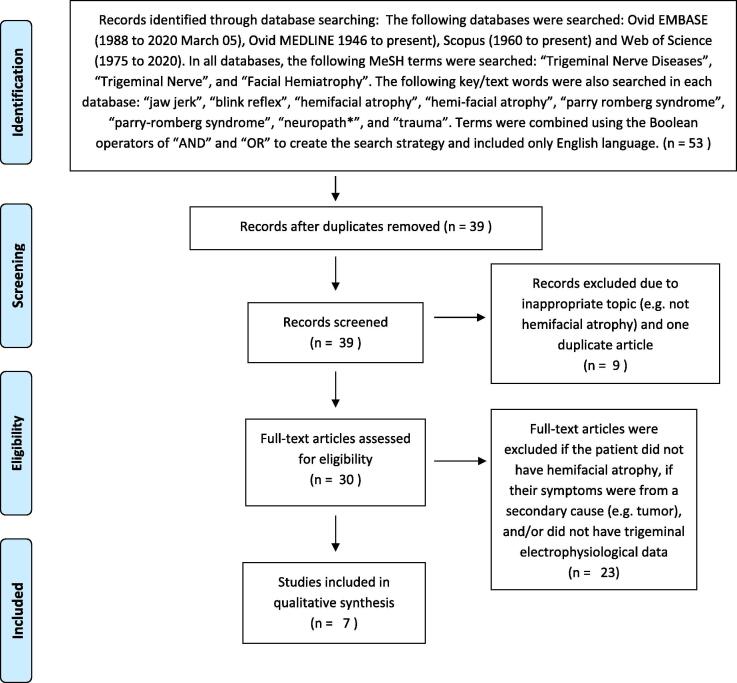
PRISMA diagram depicting the process of the systematic literature review.

In our systematic literature review, 7 references were identified for inclusion in our qualitative synthesis (Fig. 1). The articles were case reports of patient’s with HFA, and a total of 9 patients were described who also had associated trigeminal nerve electrophysiological studies (Table 1).

**Table 1:** Summary of the patient content of the seven articles identified through the PRISMA literature review.

Falla et al., *J Headache Pain.*	1	Female (40)	Right	Likely mild	Normal blink reflex, masseter inhibitory reflex, and trigeminal laser evoked potentials	n/a
Budrewicz et al., *Neurol Sci*	1	Female (26)	Right	Moderate	Abnormal blink reflexes and left trigeminal somatosensory evoked potentials	Mixed Central and Peripheral
Drumond et al., *Cephalalgia*	1	Female (32)	Right	Likely moderate	Abnormal blink reflexes	Peripheral
Lonchamp et al., *Clin Auton Res*	1	Male (14)	Right	Moderate	Abnormal blink reflexes and trigeminal somatosensory evoked potentials	Mixed Central and Peripheral
Ebersbach et al., *Mov Disord*	2	Patient 1: Male (36) Patient 2: Female (30)	Patient 1: Left Patient 2: Right	Moderate in both patients	Patient 1: abnormal needle exam of left masseter and temporalis muscles; normal masseter and blink reflexes Patient 2: abnormal needle exam of right masseter; normal masseter inhibitory and blink reflexes	Peripheral
Cruccu et al., *J Neurol Neurosurg Psychiatry*	2	Patient 1: Male (17) Patient 2: Female (50)	Patient 1: Left Patient 2: Right	Patient 1: ModeratePatient 2: Severe	Both patients: abnormal masseter inhibitory reflexes	Peripheral
Kim et al., *Arch Neurol*	1	Female (37)	Right	Moderate	Absent right masseter inhibitory reflex but normal blink reflexes	Peripheral

In our retrospective chart review, we identified 4 additional HFA patients for inclusion who had undergone trigeminal nerve electrophysiological testing. The characteristics and electrophysiological data of these 4 patients are summarized in Table 2.

**Table 2:** Retrospective case series HA patients with associated trigeminal nerve testing data.

Patient information	1	2	3	4
Sex	F	M	F	F
Age of onset	43	12	7	55
Age at testing	45	14	20	75
Side of atrophy	Right	Left	Right	Right
Severity^15^	Mild	Mild	Mild	**Moderate**
Neuro symptoms	post dental work dystonia	seizure disorder; chiari type I	seizure disorder; headaches	Ptosis
Other symptoms	scoliosis; fibromyalgia; conversion disorder	ADHD	morphea; coupe de sabre	–

Blink reflexes				
R R1 ipsi latency *(normal 8*–*13 msec)*	11.2	10.8	10.3	10.4
R R2 ipsi latency *(29*–*41 msec)*	34.9	29.8	30.6	32.5
R R2 contr latency *(≤44 msec)*	36.8	30.5	31.4	27.7
L R1 ipsi latency *(8*–*13 msec)*	11.4	10.5	10.4	10.1
L R2 ipsi latency *(29*–*41 msec)*	35.0	33.7	31	35.9
L R2 contra latency *(≤44 msec)*	37.2	34.0	31	35.6

Nerve conduction studies				
R facial motor amp *(>1.8 milli/micro volts)*	2.6	2.1	3.1	2.5
R facial motor latency *(<4.1 millisec)*	3.1	3.1	2.8	3.0
L facial motor amp *(>1.8 milli/micro volts)*	3.0	2.0	3.3	3.6
L facial motor latency *(<4.1 millisec)*	3.0	3.6	2.8	3.0

EMG needle exam				
Temporalis, ipsilateral	Normal	–	–	**Neurogenic:** Long duration, polyphasic motor unit potentials
Masseter, ipsilateral	Normal	Normal	–	**Neurogenic:** Long duration, polyphasic motor unit potentials

Overall, of the 13 total HFA patients identified, 9 (69%) were female with a median age at the time of testing of 30 years (range 14–55). At the time of testing, the patients had HFA severity characterized as mild (n = 4), moderate (n = 8), and severe (n = 1). Testing included: bilateral blink reflexes (n = 11), masseter inhibitory reflexes (n = 6), trigeminal laser evoked potentials (n = 1), trigeminal somatosensory evoked potentials (n = 2), and needle EMG including testing of the masseter muscle (n = 6).

Trigeminal nerve testing was abnormal in 9/13 (69%) cases, with abnormalities only seen in moderate-severe disease. Abnormal results most often indicated a peripheral localization (7/9, 78%), but a mixed central and peripheral localization (2/9, 22%) was suggested in a minority. Of the 4 HFA cases with normal electrophysiological testing, all were classifiable as having a mild form.

## 4 Discussion and conclusions

In our systematic literature review and retrospective case series, we provide a novel assessment of the role of trigeminal nerve electrophysiological testing in HFA. Our results show that while electrophysiological abnormalities are seen in approximately three fourths of patients, these changes appear to be limited to those with moderate-severe disease progression where muscle atrophy is characteristic. We did not identify trigeminal electrophysiological abnormalities in those with mild disease. Therefore, in contrast to our hypothesis, trigeminal nerve pathology would appear to be most likely a consequence as opposed to the cause of HFA. Based on this available experience, trigeminal nerve electrophysiology could potentially serve as a biomarker for more advanced disease progression but would not be anticipated to serve a role in early recognition and diagnosis of mild cases.

An additional observation from our study is that trigeminal pathophysiology appears to most commonly involve the peripheral nerve, with mixed central and peripheral abnormalities being a more uncommon exception. This finding would be consistent with involvement as part of the progressive, segmental degeneration observed clinically. However, patients in our series were not evaluated in an identical protocolized fashion, and those with a central localization additionally underwent evoked potential studies, which the others did not. Therefore, the identification of central findings may have been inadequately screened for in our cohort, and may be under-recognized based on the available results.

Indeed, whether the cause of HFA is from a disorder of the central versus peripheral nervous system has been hotly debated in the literature. Studies that support a central cause point out the association of HFA with epilepsy as a potential indicator of central nervous system dysfunction (Lonchampt et al., 1995, Budrewicz et al., 2012). Another study supportive of a central etiology suggests the pathogenesis is in the autonomic nervous system (Wartenburg, 1945). In this putative mechanism, the sympathetic nervous system stimulates the atrophy of the fat and subcutaneous tissues largely mediated through adrenergic receptors and results in the clinical phenotypes seen in HFA including mydriasis, hyperpigmentation, and hyperhidrosis. In contrast, other studies suggest that HFA affects primarily the peripheral nervous system citing associated clinical features such as hemimasticatory spasm (Ebersbach et al., 1995). The leading suspicion is for a compressive or focal demyelinating condition that could result in atrophy, spasms, or often both of these conditions (Cruccu et al., 1994). More research fleshing out these two potential causes is needed.

Limitations to the current study include a small sample size and the challenges inherent to a retrospective study design. The disease is rare and occurs in approximately 1:700,000 patients (Bucher et al., 2016). Thus, despite these limitations, this clinical and electrophysiologic review of 13 patients is a useful opportunity to glean insights into the pathophysiology of HFA.

The current study adds to the literature in reporting that trigeminal nerve abnormalities appear to be common in HFA, and appear to be limited to those with moderate-severe disease progression. Peripheral trigeminal involvement is most often reported, although mixed central and peripheral causes are also seen and may represent a distinct clinical entity. Future work, especially if disease-modifying therapies become available, should consider the role of trigeminal electrophysiology in disease monitoring for moderate disease progression among those with skin involvement alone.

## Conflict of Interest Statement

None of the authors have potential conflicts of interest to be disclosed.
